# Metabolomic Insights into Prostate Cancer Treatment and Relapse

**DOI:** 10.3390/cancers17243993

**Published:** 2025-12-15

**Authors:** Kristina Lundquist, Henrik Antti, Camilla Thellenberg Karlsson

**Affiliations:** 1 Department of Chemistry, Umeå University, 901 87 Umeå, Sweden; henrik.antti@umu.se; 2 Department of Diagnostics and Intervention, Umeå University, 901 87 Umeå, Sweden; camilla.thellenberg@umu.se

**Keywords:** prostate cancer, metabolomics, hormone therapy, radiotherapy, chemometrics, cholesterol

## Abstract

**Background**: High-risk prostate cancer is often treated with combined androgen deprivation therapy (ADT) and radiotherapy (RT). Blood biomarkers may enable treatments to be tailored to individual patients. Metabolomics, the study of small-molecule alterations in blood, is promising, and lipids are emerging as potential markers of poor prognosis. This study aims to investigate metabolic changes during prostate cancer treatment and their correlation to disease outcome. **Methods**: This study included 136 blood plasma samples from 35 patients with high-risk prostate cancer treated with RT and ADT, recruited from the Uppsala/Umeå Comprehensive Cancer Consortium (U-CAN) project. Blood samples were collected before, during, and after treatment and analyzed at Metabolon Inc. (Durham, NC, USA). To study differences in metabolic levels during treatment, three different sampling time points were considered: before ADT, in-between ADT and RT, and after RT. Both multivariate (orthogonal projections to latent structures, OPLS) and univariate analyses were performed, where statistical significance in combination with a large fold change was considered indicative of a substantial change. **Results**: Significant changes in metabolite levels were observed. Many of the significant metabolites for the whole course of treatment were also significant during ADT but not during RT, indicating that changes during ADT dominated the overall treatment. Changes were found to be especially common in steroids and fatty acids. Multivariate analysis revealed significant differences in metabolites between relapsing and non-relapsing patients. Among the significant metabolites were cholesterol and epiandrosterone. **Conclusions**: Metabolomics can identify biomarkers for prostate cancer treatment response and relapse. Further studies are needed to identify patterns and individual metabolites to personalize treatment strategies for prostate cancer.

## Simple Summary

Prostate cancer is one of the most common types of cancer, and personalized treatment strategies—particularly for patients with a poor prognosis—are needed. A promising approach to making treatment decisions involves identifying biomarkers in blood. In our study, we investigated metabolic changes in the blood of patients with high-risk prostate cancer and examined how these changes relate to disease progression and treatment outcomes. We observed clear evidence of metabolic alterations during treatment. While some of the affected biomarkers have been previously reported, we also identified several novel potential markers. Importantly, we found specific biomarkers that were significantly associated with disease outcomes. One of these was cholesterol, a molecule that has been extensively studied in this context. In summary, our findings highlight several promising biomarkers that may contribute to improved understanding and management of high-risk prostate cancer. Further research is needed to validate these results and explore their clinical potential.

## 1. Introduction

High-risk prostate cancer patients are often treated with a combination of therapies consisting of androgen deprivation therapy (ADT) and radiotherapy (RT) to the prostate. In recent years, a new approach was developed, which involves radiotherapy to lymph node stations [1] in the pelvis and intensified hormonal treatment with the addition of abiraterone to ADT. This approach has become standard for very-high-risk cases [2]. Risk classification tools are being developed using artificial intelligence based on ordinary histopathology in conjunction with clinical data [3] but have yet to prove useful for differentiating which treatment best suits which patient. Biomarkers in different forms might be helpful to choose which intensification will best help the individual patient.

The prospect of using easily accessible biomarkers in blood to obtain earlier indication of individual treatment outcome is of high relevance to further increase the possibilities for an individualized treatment with improved long-term outcome. Metabolomics, an omics technique offering the option to detect individual or patterns of small molecules in blood and other biosamples, has been proven to provide predictive information regarding treatment response in prostate cancer [4]. Lipids are emerging as a possible marker for poor prognosis [5,6] and are within the scope of metabolomics.

In the life sciences, metabolomics has become an important complement to other, more mature omics techniques, e.g., genomics and proteomics [7,8,9,10]. The complex multivariate data generated in metabolomics studies requires a customized bioinformatics approach and set of tools. These are provided by means of chemometrics, which include approaches such as data exploration, pattern recognition, and independent and dependent multivariate statistical analysis [11]. The main benefits of the chemometrics methods for multivariate analysis, e.g., partial least squares (PLS) and orthogonal projections to latent structures (OPLS)-based methods, are their ability to form robust and interpretable models that can extract and validate biomarker patterns for further prediction (diagnosis/prognosis) or mechanistic interpretation (pathway analysis).

Key to providing biomarkers or biomarker patterns of potential clinical use is the combination of a clinically relevant study design, with a method for sensitive metabolite detection and bioinformatics tools that can extract information from complex multivariate data. This is offered by a purpose-designed sequential application of metabolomics and chemometric methods, as suggested in the present study. Here, we aim to investigate metabolic changes during the treatment of prostate cancer and to further study how metabolic changes after treatment correlate with disease outcome.

## 2. Materials and Methods

### 2.1. Study Participants

Study participants were recruited from the Uppsala/Umeå Comprehensive Cancer Consortium (U-CAN) project [12]. Included patients had been diagnosed with high-risk prostate cancer, with either high PSA, a high Gleason score or T3 status, or a combination thereof. All participants received three months of neo-adjuvant androgen deprivation therapy (ADT) with subsequent conventionally fractionated radiotherapy (RT) followed by adjuvant ADT for six months. The group that was selected is part of a larger project were proteomics and analysis of immune responses were also performed [13]. Blood samples were taken at different time points during treatment (Supplementary Figure S1) and stored at −80 °C within two hours from blood draw. In total, 136 blood plasma samples from 35 unique patients were included in the study, and 31 of those had a follow-up sample taken after end of treatment (median = 7 months). The study participants were included in U-CAN during 2013–2015 and followed up to determine clinical outcomes as of 31 December 2023, as summarized in Table 1 and Supplementary Figure S2. In short, fifteen of the study participants experienced PSA relapse (43%), twelve developed metastases, four died of prostate cancer, and another four died of other causes.

### 2.2. Metabolomics Analysis and Preprocessing

Metabolomics analysis of the blood samples was performed with UPLC-MS/MS (Ultrahigh Performance Liquid Chromatography–Tandem Mass Spectroscopy) at Metabolon Inc. (Durham, NC, USA). A detailed description of the analysis procedure can be found in [14]. In this study, we employed a constrained randomized run order [15], where samples from a specific patient were constrained to the same batch to minimize batch-effects, i.e., variation due to differences in batches.

Out of 1225 metabolites detected by Metabolon, 754–766 were retained for further analysis, depending on the model (see “Statistical analysis and models”). Excluded metabolites were either unidentified, only partially characterized, known drug compounds, or metabolites with too many missing values (≥20%). Missing values in the remaining metabolites were imputed with a value of half the minimal value for that specific metabolite. Since our experimental design minimized batch effects in samples originating from the same patient, no batch normalization was performed for the comparisons of samples during treatment. However, samples from relapsing patients and samples from non-relapsing patients were run in multiple batches, and for this comparison, the raw data was batch-normalized, i.e., the metabolite values were divided by the median of each batch. Finally, for all comparisons, values were log-transformed prior to statistical analysis. In summary, the raw data obtained from analysis, was pre-processed by first excluding metabolites according to the criteria mentioned above, and thereafter imputing missing values. A batch-normalization was performed when comparing samples from relapsing patients with samples from non-relapsing patients, but not for the comparisons during treatment. Subsequently, all values were log-transformed.

### 2.3. Statistical Analysis and Models

Differences in metabolic levels during treatment were studied by making three different comparisons. Firstly, changes during ADT were assessed by comparing samples taken before treatment (first sample) with samples taken between ADT and RT (second sample), the so-called ADT model. Secondly, changes during RT were investigated by comparing the “in-between” samples (second sample), with samples taken after the whole treatment (third sample), i.e., the RT model. Finally, to analyze changes during the whole course of treatment, samples before treatment (first sample) were compared with samples after treatment (third sample), forming the TT model (TT = Total Treatment).

Multivariate analysis was performed in SIMCA (version 17.0, Sartorius Stedim Data Analytics AB). Analysis of paired differences between consecutive samples during treatment was achieved by means of OPLS–Effect Projection (OPLS—EP) [15]. To investigate metabolic changes between relapsing and non-relapsing patients, both Principal Component Analysis (PCA) [16] and OPLS–Discriminant Analysis (OPLS-DA) [17] were performed. Firstly, to obtain an overview of the metabolic changes in the data, four different PCA models were calculated based on samples taken at four different time points (three samples during treatment, as described above, and one follow-up sample). To further investigate the separation of the two patient groups, an OPLS-DA model based on the follow-up samples was performed. To evaluate the performance of the multivariate models, the *p*-value from ANOVA of the cross-validated model residuals (CV-ANOVA) [18], R^2^ (the coefficient of determination), and Q^2^ (predictive ability) were obtained.

Univariate analysis was performed in R (v4.1.3; R Core Team 2022), where individual metabolic changes were illustrated by volcano plots. In the volcano plots, *p*-values from a two-sided paired *t*-test (ADT, RT, and TT models) or Student’s *t*-test (relapsing versus non-relapsing) were combined with calculated fold change (FC) values. For the models comparing differences during treatment, FC was calculated as the mean value for samples taken after treatment divided by the mean value for samples taken before treatment. Thus, an FC below 1 indicates lower values after treatment, and vice versa, an FC > 1 indicates higher levels after treatment. Similarly, for comparing relapsing patients with non-relapsing patients, FC was calculated as the mean value for samples taken from relapsing patients divided by the mean value for samples taken from non-relapsing patients. Thus, an FC above 1 indicates higher values for relapsing patients. To address the problem with multiple test comparisons the *p*-values were subjected to false discovery rate (FDR) correction [19]. We considered both statistical significances, i.e., FDR-corrected *p*-value of less than 0.05, and an FC above 1.5 or below 0.67, i.e., 3/2 or 2/3, to be indicative of a substantial change in metabolic levels.

All figures were created in R (v4.1.3; R Core Team 2022). The two different *t*-tests as well as FDR-correction was performed in base R with the following functions: *t.test* and *p.adjust*. The function *p.adjust* recalculates the *p*-values into so called q-values.

## 3. Results

### 3.1. Changes in Metabolic Levels During Treatment of Prostate Cancer

**Figure 1:**
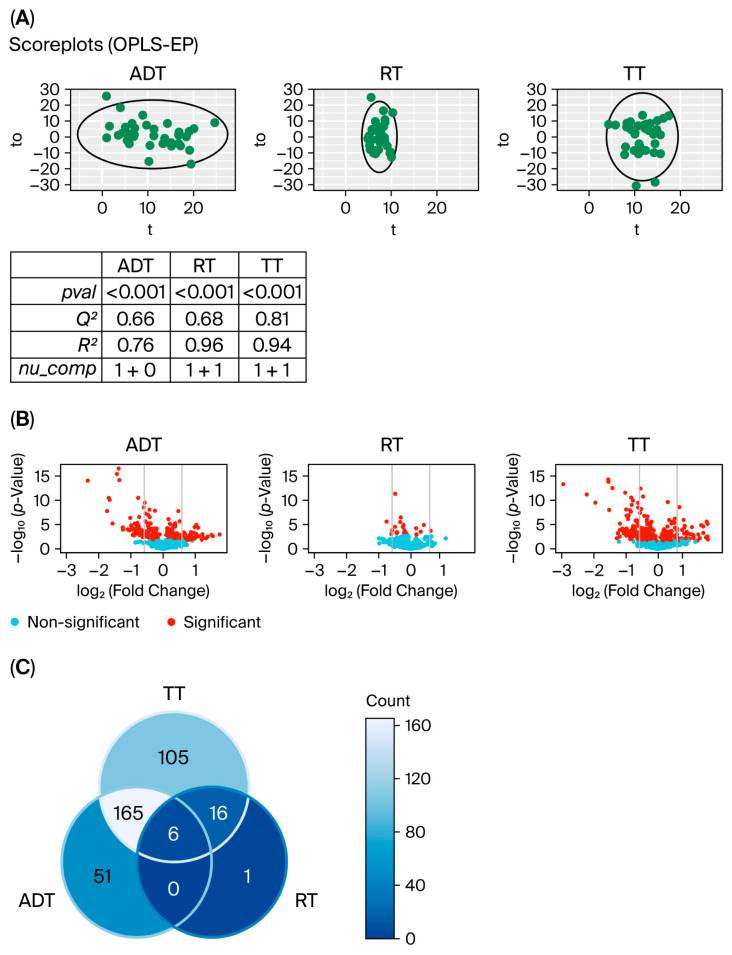
Paired comparison between samples taken at different time points during treatment, i.e., during androgen deprivation therapy (ADT), radiotherapy (RT), and for the whole treatment course (TT). (**A**) Score plots from the OPLS—EP (Orthogonal Projections to Latent Structures–Effect Projection) models show that the largest variation was in the ADT model and the smallest in the RT model. Ellipse: Hotelling’s T2 (95%). All models are significant and show high Q^2^ and R^2^ values, as seen in the table. (**B**) Volcano plots showing significant metabolites in red and vertical lines indicate a fold change of 1.5 or 0.67, i.e., |log_2_(FC)| = 0.6. The number of significant metabolites is considerably smaller in the RT model compared to the two other models. (**C**) The Venn diagram shows the number of significant metabolites shared between the three models. Many of the significant metabolites are shared between the ADT and TT models (*n* = 171, 50%). In summary, (**A**–**C**) show that the ADT model shares many features with the TT model, whereas the RT model is different from the other two.

Multivariate analyses showed significant overall changes in metabolite levels during the treatment of prostate cancer patients. In accordance with the different treatment stages, three different multivariate models were constructed using OPLS-EP, i.e., the ADT, RT, and TT model (as described in the Materials and Methods section). All OPLS-EP models showed significant alterations in the metabolite profile (*p*-value < 0.001) and performed well based on their Q^2^ and R^2^ values (Figure 1A). Metabolic changes were further investigated in three different volcano plots, one for each model (Figure 1B). Comparing the three volcano plots revealed a similar pattern for the ADT and TT models, and demonstrated that the RT model is different from the other two models. The number of significant metabolites was considerably larger in the ADT (*n* = 222) and TT models (*n* = 292) compared to the RT model (*n* = 23) (Figure 1B,C). In addition, only three (13%) of the significant metabolites in the RT model had an FC above 1.5 or below 0.67, in comparison to 92 (41%) in the ADT model and 122 (42%) in the TT model. Many of the total numbers of significant metabolites were shared between the ADT and TT models (*n* = 171, 50%), but only a few were shared between the RT and TT models (*n* = 22, 6%) (Figure 1C). Thus, overall changes during the whole course of treatment were dominated by changes during ADT. In addition, the direction of change for the significant metabolites, i.e., down- or upregulation, was the same for the ADT and TT models, and the overall changes persisted even after treatment.

Significant metabolites that also have an FC > 1.5 or an FC < 0.67 are listed in Supplementary Table S1. Of the 140 metabolites listed, 88 (63%) were downregulated, and of these, 23 were steroids and 54 fatty acids. Of all the downregulated metabolites with the lowest *p*-value, many were steroids, e.g., the 20 with the lowest *p*-value 17 (85%) were steroids. Some of the listed metabolites have been found by others to have a link with prostate cancer [20,21,22,23]; however, some were newly discovered by our study. A subset of the 140 metabolites listed in Supplementary Table S1, i.e., those with smallest *p*-value, are displayed as heatmaps in Supplementary Figure S3 for the three different models, respectively.

### 3.2. Comparing Changes in Metabolic Levels for Patients Who Relapse with Those That Do Not

**Figure 2:**
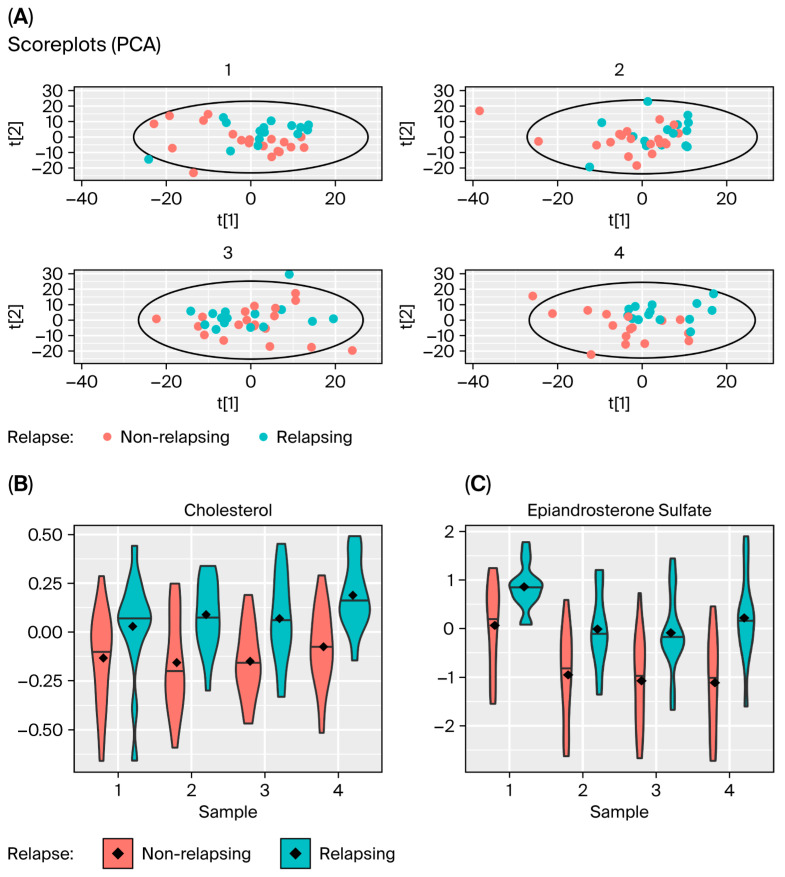
Comparison of samples taken from relapsing (turquoise) and non-relapsing (red) patients. Samples were taken before treatment (1), in between treatments (2), after treatment (3), and from follow-up (4). (**A**) Score plots from Principal Component Analysis (PCA) based on metabolic levels from samples taken at the four different sampling time points (1–4) show a trend towards larger group separation over time, i.e., from 1 to 4. Ellipse: Hotelling’s T2 (95%). (**B**,**C**) Violin plots showing levels of two specific metabolites, cholesterol (**B**) and epiandrosterone sulfate (**C**), comparing the two patient groups at the four different sampling time points (1–4). The horizontal lines inside the violin plots indicate median values, and the diamonds indicate mean values. Metabolic levels for the relapsing patients are noticeably higher compared to those for the non-relapsing patients. Cholesterol levels (**B**) are stable from the start to end of treatment and increase during follow-up. This pattern is, however, not seen for epiandrosterone sulfate (**C**), where the relative high levels seen before the start of treatment drop during treatment and then increase during follow-up for the relapsing patients but not for the non-relapsing patients.

PCA modeling revealed metabolic differences between samples from relapsing patients compared with samples from non-relapsing patients (Figure 2A). There was a noticeable trend towards more separation of the two groups as the treatment progressed (one to four in Figure 2A), with an evident separation in the follow-up samples (4). Subsequent OPLS-DA modeling based only on the follow-up samples showed a clear significant difference between relapsing and non-relapsing patients (*p* ≈ 0.001, R^2^ ≈ 0.7, Q^2^ ≈ 0.4). OPLS-DA models for the other three time points were not significant, and score plots for all four different OPLS-DA models are shown in Supplementary Figure S4.

In total, 16 of the metabolites were significant (all upregulated), and of these, 9 also showed an FC > 1.5 (Supplementary Figure S5 and Supplementary Table S2). Further investigation of the changes in metabolic level revealed that most of the 16 significant metabolites showed the same pattern: smaller changes already existed in samples at diagnosis and during the treatment process. This was exemplified for cholesterol (Figure 2B). The only exception to this pattern was the only significant steroid, epiandrosterone sulfate. Here, the levels went down during treatment and back up again in the follow-up sample for the relapsing but not for the non-relapsing patients (Figure 2C). One of the significant metabolites, cholesterol, has been thoroughly studied previously in connection with the treatment and recurrence of prostate cancer. In this study, the cholesterol levels were consistently higher for those patients that relapsed compared with those that did not, and the follow-up sample exhibited a larger difference than the other samples (Figure 2B). All the metabolites obtained from Metabolon are listed in Supplementary Table S3.

## 4. Discussion

Our study revealed significant metabolic changes in blood, particularly in steroids and fatty acids, during prostate cancer treatment, with many metabolites showing lower levels after treatment. We have previously shown how the use of multivariate paired analysis by means of OPLS-EP [15] increases the signal-to-noise ratio in data when the study samples have a paired structure, making it a more sensitive tool for detecting changes in metabolite profiles over time. Combining these approaches in this study revealed stronger and novel metabolic changes related to prostate cancer treatment compared to previous studies. Notably, we identified 16 significant metabolites, including cholesterol and epiandrostendione, which were consistently higher in the blood of patients who relapsed.

Cholesterol has previously been shown to increase after initiating ADT [24] and to be directly regulated by androgens [25], as well as several other hallmarks of the metabolic syndrome [26]. The presence of elevated blood cholesterol has been identified as a risk factor for recurrence among men undergoing prostatectomy where no ADT was used [27]. In addition, elevated cholesterol levels have been found in the bone metastases of patients with prostate cancer compared to patients with normal bone or bone metastases from other types of cancer [28]. Since there is ample evidence that lipids, especially cholesterol, play an important part in prostate cancer, epidemiological studies have investigated the use of statins in relation to the outcome of prostate cancer. Two meta-analyses have found a decreased recurrence of disease following radiotherapy but not after radical prostatectomy in patients treated with statins [29,30].

In post hoc analyses of two large, randomized trials of the combination of ADT and abiraterone where the use of statin was recorded, there was a clear benefit for the patients using statins, especially those who also received abiraterone [31]. One hypothesis is that statins may synergize with ADT by reducing intratumoral cholesterol, reducing the substrate for de novo androgen synthesis. The finding in our study of elevated cholesterol and epiandrostenedione, both precursors for testosterone, among the patients who later relapsed may indicate that these are the patients in most need of treatment intensification with the addition of abiraterone to radiotherapy and ADT. This warrants further study in a larger patient cohort, preferably a clinical trial.

**Figure 3:**
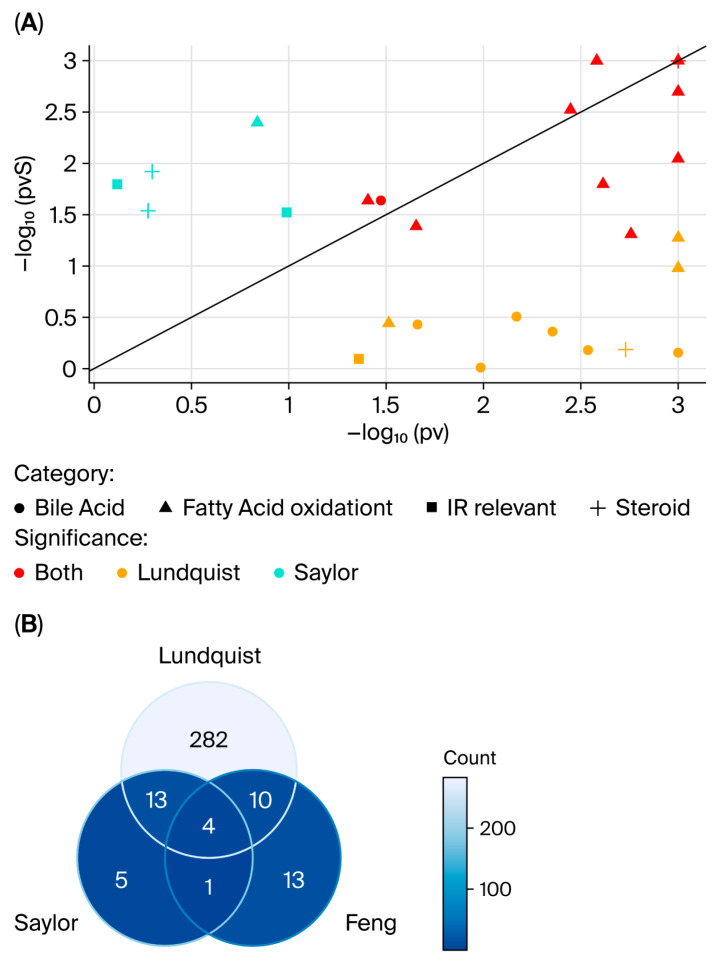
Comparison of our results with results from two other studies: Saylor et al. [17] (**A**,**B**) and Feng et al. [16] (**B**). (**A**) Scatter plot of log-transformed *p*-values from the ADT model (*x*-axis) versus log-transformed *p*-values from [17] (*y*-axis). Significant metabolites in both studies (*p* < 0.05) are colored in red. Metabolites that were significant in our study but not in [17] are colored yellow, and conversely, metabolites that were significant in [17] but not in our study are colored turquoise. Note that for comparison reasons, all *p*-values less than 0.001 are set to 0.001 (=3 in the plot). The line represents values that are equal in the two studies. (**B**) Venn diagram comparing significant metabolites in the three studies (Lundquist et al., Saylor et al. [17], and Feng et al. [16]). Most of the metabolites that were significant in Saylor et al.’s study were also significant in our study, and more than half of the significant metabolites in Feng et al.’s [16] study were also significant in our study. In addition, a large portion of metabolites that were significant in our study (90%) were not found in the two other studies, which might be due to a larger number of identified metabolites or differences in pre-processing (e.g., imputation) or a combination thereof.

Our findings align with previous studies, such as those by Saylor et al. [23] and Feng et al. [21], who reported similar metabolic changes in blood during ADT. For example, Saylor et al. found that after ADT, steroid levels decreased, bile acids increased, lipid metabolism decreased, and markers of insulin resistance were lower/stable. Our study confirmed and, in some cases, strengthened these results (Figure 3A). For example, some of the bile acids reported by Saylor as weak differences show stronger differences in our study (yellow circles). However, the two significant markers of insulin resistance (IR) in their study did not show a significant difference in our study (turquoise squares). Most metabolites were found to have the same direction of change, i.e., down-/upregulation, in both studies, and for those metabolites that did have an altered direction of change, the FC was close to 1 in both studies. Our study also showed alignment with the work of Feng et al. [21]. Notably, we identified several additional metabolites of interest (Figure 3B) that were not reported in the two previous studies. The larger number of unique, significant metabolites observed in our study may be attributed to the greater number of metabolites identified than in earlier studies, or to differences in data pre-processing—particularly the imputation step. Since we imputed half the minimum value instead of the full minimum value, we may have introduced artificially large differences for metabolic levels.

One limitation of our study is the limited number of patients and samples, which warrants caution in interpreting our results onto a broader population. However, our results align with previous studies and are promising for further studies and validation.

Another limitation of our study is the lack of adjustment for confounders such as clinical factors, BMI, co-morbidities, and medications other than the ones used for treating the prostate cancer. Other limitations of our study include the non-fasting blood samples and lack of information on additional medications, which could influence the results. The difference in levels of cholesterol early on in the disease course could indicate a difference in disease characteristics or be due to treatment with statins. However, it is unlikely that non-cancer treatments would consistently differ between relapsing and non-relapsing patients.

Using serial samples from the same patients allowed us to capture metabolite changes at different time points during treatment and recovery. This setup helps illuminate metabolic triggers of treatment, reveals the underlying mechanisms of treatment response, and can give some insights into patient relapses. We have not performed functional validation of our findings nor validation within a larger cohort of patients, something we will aim to address further on.

## 5. Conclusions

This study revealed significant metabolic changes in blood during high-risk prostate cancer treatment, identifying mostly steroids and fatty acids as the most affected metabolites. In addition, metabolic differences between relapsing and non-relapsing patients were clearly identified. For cholesterol, there seemed to be a difference early, before any treatment was administered, maybe indicating a difference in the nature of the cancer. These differences warrant further investigation, especially since one of the metabolites is the well-known cholesterol that can be targeted by statins.

## Figures and Tables

**Table 1:** Clinical characteristics for patients grouped according to outcome events.

	All (*n* = 35)	Relapse (*n* = 15)	Metastasis (*n* = 12)	Deceased (*n* = 8)	Deceased, PCa (*n* = 4)
**Age** **(years)**	58–79	58–79	58–79	61–75	61–75
**T-stage**					
T1	3	1	0	0	0
T2	13	6	6	3	2
T3	17	6	4	4	1
T4	2	2	2	1	1
***N *** **-stage**					
N0	30	17	9	6	3
N1	5	3	3	2	1
**ISUP**					
1	3	1	0	1	3
2	5	1	1	2	0
3	8	3	2	1	1
4	2	0	0	0	0
5	17	10	9	4	3
**Time to event (years) ** ^1^	9.0 (1.6–9.5)	3.3 (1.3–8.2)	4.1 (2.4–6.1)	6.3 (1.6–8.3)	6.8 (6.2–8.3)
**PSA at diagnosis/relapse ** ^1^	30.0 (6.7–232)	2.9 (2.1–8.6)			

^1^ Median (range).

## Data Availability

The data presented in this study are only available on request from the corresponding author due to GDPR and sensitive patient data.

## Supplementary Materials

The following supporting information can be downloaded at https://www.mdpi.com/article/10.3390/cancers17243993/s1: Figure S1: Timeline of treatment and sampling points; Figure S2: Schematic overview of the number of patients that experience different events during a period of up to 10 years after start of treatment; Figure S3: Heatmaps over the metabolites with FDR-corrected *p*-value < 0.001 grouped according to whether if the patient relapsed or not; Figure S4: Scoreplots from OPLS-DA comparing samples taken from relapsing (green) and non-relapsing (blue) patients; Figure S5: Volcano plot based on differences in metabolic levels between relapsing and non-relapsing patients for the follow-up samples; Table S1: Interesting metabolites (*n* = 140) during treatment; Table S1: Significant metabolites with a FC > 1.5 or FC < 0.67 (*n* = 140); Table S2: Significant metabolites (*n* = 16), for the comparison of samples from relapsing and non-relapsing patients; Table S3: List of all metabolites before exclusion.

## Abbreviations

The following abbreviations are used in this manuscript:

ADT	Androgen Deprivation Therapy
RT	Radiotherapy
UPLC-MS/MS	Ultra-Performance Liquid Chromatography–Tandem Mass Spectrometry
PCA	Principal Component Analysis
PLS	Partial Least Squares
OPLS	Orthogonal Projections to Latent Structures
OPLS-EP	OPLS–Effect Projection
OPLS-DA	OPLS–Discriminant Analysis
CV-ANOVA	Cross-Validated ANOVA
FC	Fold Change
FDR	False Discovery Rate
