# Supplementary material for: Metabolomic Insights into Prostate Cancer Treatment and Relapse
Source: Cancers (Basel). 2025 Dec 15;17(24):3993. doi: 10.3390/cancers17243993 (PMC12730953; doi:10.3390/cancers17243993)
Supplement: Supplementary file 1 [file cancers-17-03993-s001.zip › SupplementaryFigures_revised.pdf]

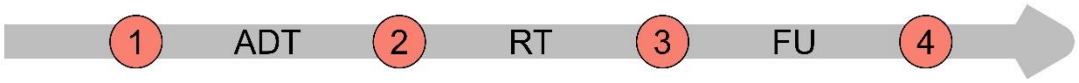

Supplementary Figure S1. Timeline of treatment and sampling points. The consecutive treatments are Androgen Deprivation therapy (ADT) and radiotherapy (RT) respectively, followed by a follow-up period (FU), with a median time period of 7 months. Sampling is indicated by red circles and blood samples are drawn at the following timepoints: before ADT (1), in between ADT and RT (2), after RT (3) and after the follow-up period (4).

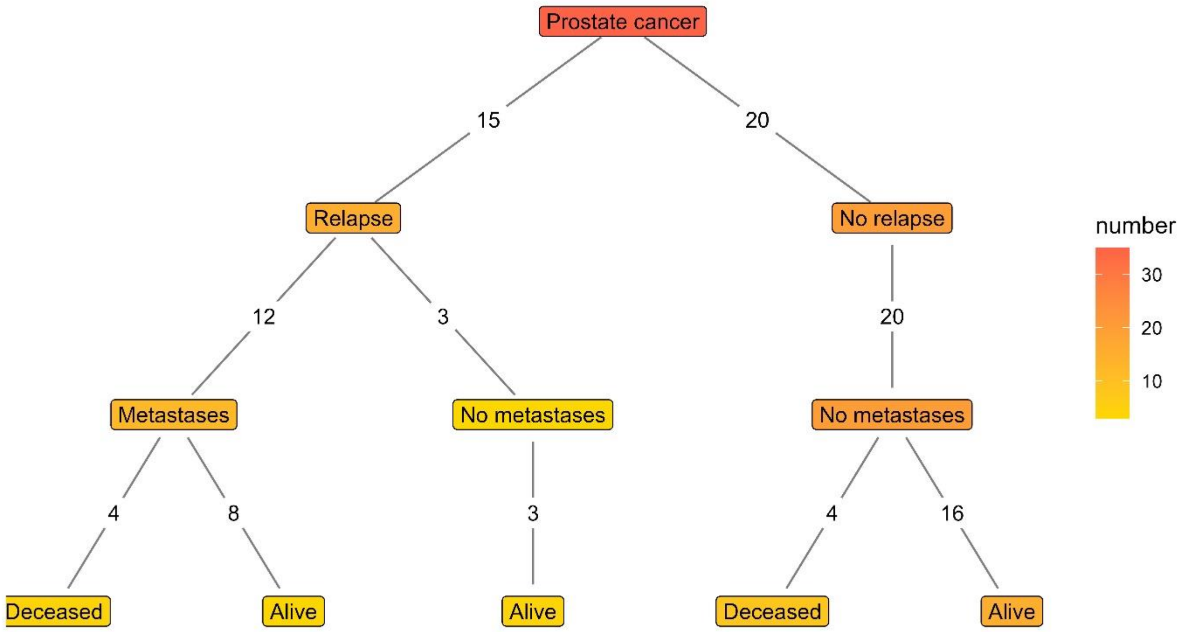

Supplementary Figure S2. Schematic overview of the number of patients that experience different events during a follow-up period of up to 10 years after start of treatment. The study involves 35 prostate cancer patients, of which 15 relapsed and 20 did not. Of the 15 that relapsed 12 developed metastases and four died.

A

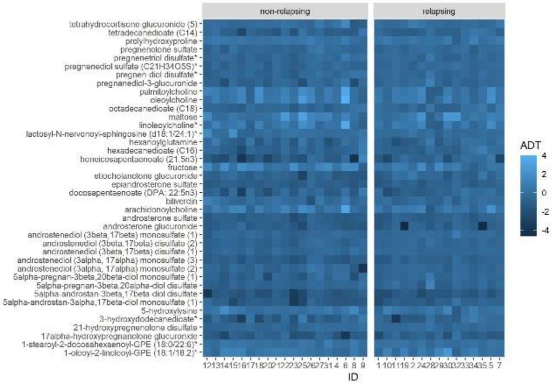

B

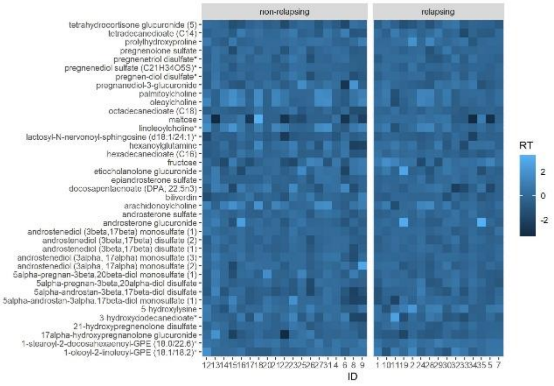

C

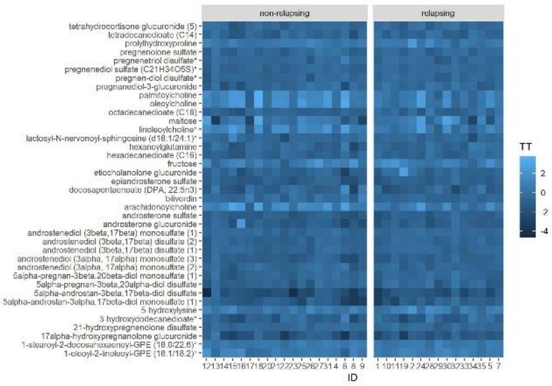

Supplementary Figure S3. Heatmaps over the metabolites with FDR-corrected p-value < 0.001 (see Supplementary Table 1) grouped according to whether if the patient relapsed or not. **(A)** Heatmap for differences in metabolic values between samples taken before and after ADT. **(B)** Heatmap for differences in metabolic values between samples taken before and after RT. **(C)** Heatmap for differences in metabolic values between samples taken before and after the whole course of treatment.

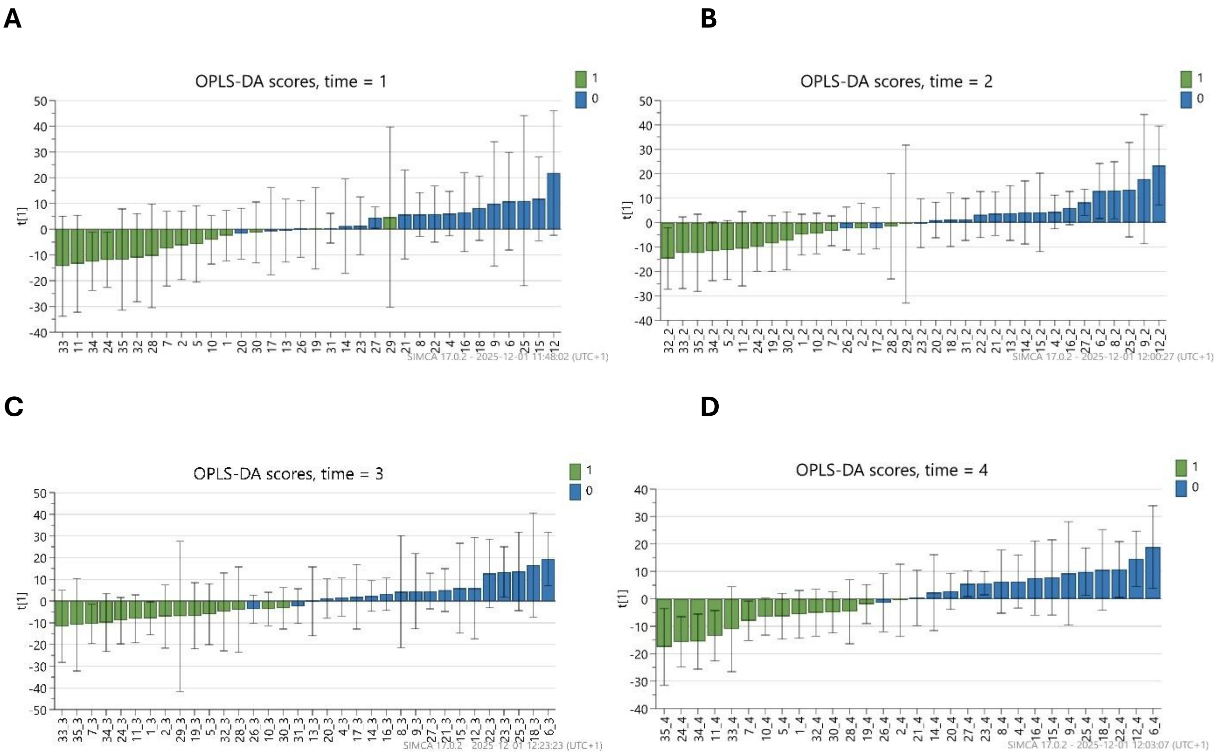

Supplementary Figure S4. Comparison of samples taken from relapsing (green) and non-relapsing (blue) patients. The figure shows scoreplots from OPLS-DA based on metabolic levels from samples taken before treatment (A), in between treatments (B), after treatment (C) and from follow-up (D) respectively. Only the model for the follow-up samples is significant ($p < 0.05$) and all models show small to moderate Q^2- and R^2-values with the model for the follow-up samples having the largest values. The Q^2-values range between -0.1 and 0.4 and the R^2 values range between 0.6 and 0.7.

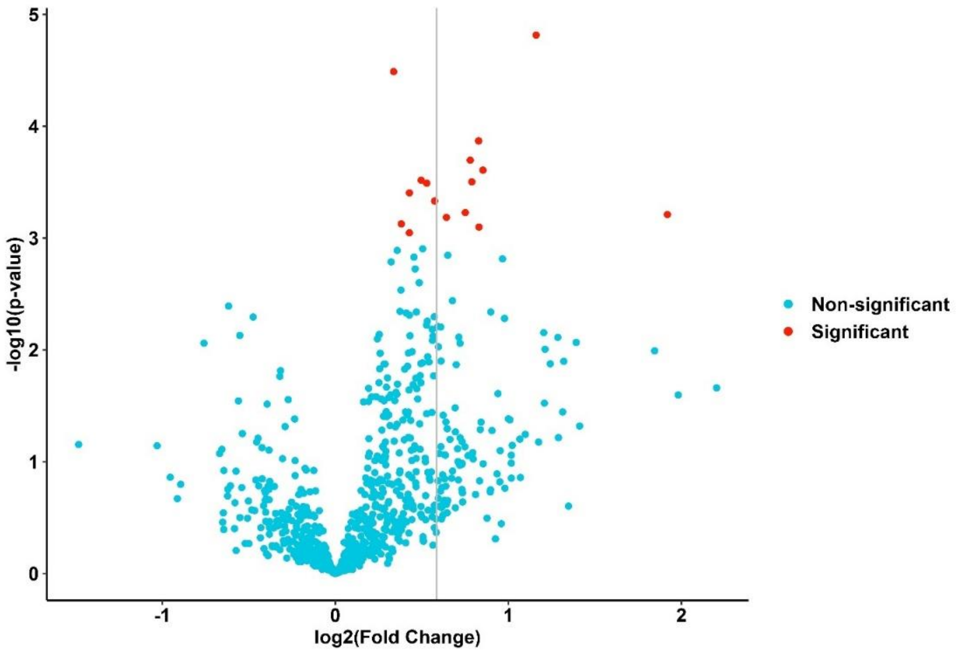

Supplementary Figure S5. Volcano plot based on differences in metabolic levels between relapsing and non-relapsing patients for the follow-up samples. Significant metabolites are shown in red, and the vertical line indicates log2(FC) = 0.6, i.e. a fold change of 1.5. In total 16 metabolites are significant (listed in Supplementary Table S2) and nine of those have a fold change above 1.5.
